# Hepatic Portal Venous Gas Incidentally Associated With Bacterial Enteritis: A Case Report

**DOI:** 10.7759/cureus.10219

**Published:** 2020-09-03

**Authors:** Brian Sumner, William Bonadio, Christopher Hahn

**Affiliations:** 1 Department of Emergency Medicine, Mount Sinai Morningside, New York, USA; 2 Department of Pediatric Emergency Medicine, Mount Sinai Morningside, New York, USA

**Keywords:** epec, gastroenteritis, portal venous gas, e. coli, pediatric emergency medicine

## Abstract

Hepatic portal venous gas (HPVG) has long been associated with catastrophic intraabdominal conditions. Advancements in ultrasound (US) and computed tomography (CT) imaging have resulted in an increased number of incidental and clinically benign HPVG cases identified. Causes of clinically benign HPVG include viral gastroenteritis, gastritis, pancreatitis, appendicitis and diverticulitis. Our case demonstrates the first reported case of HPVG in an adolescent patient associated with enteropathogenic E. coli (EPEC). The patient’s course was favorable, marked by a short stay in the pediatric intensive care unit (ICU) and did not require surgical intervention. With higher sensitivity of imaging modalities to diagnose both suspected and incidental cases of HPVG, clinicians will be required to consider the risks and benefits of conservative treatment or surgical intervention.

## Introduction

In 1955 Wolfe and Evans described hepatic portal venous gas (HPVG) on plain abdominal radiography in newborns with necrotizing enterocolitis [1]. Since then the detection of HPVG has been largely associated with catastrophic intraabdominal conditions and associated mortality rates reported as high as 75% [2]. With the advent of advanced imaging such as US and CT scan, detection rates have notably increased and expanded the differential diagnosis associated with HPVG [3]. Some clinically benign causes of HPVG include gastritis, pancreatitis, appendicitis, diverticulitis and viral gastroenteritis [2-6]. 

We describe, to our knowledge, the first reported case of a patient presenting with EPEC confirmed gastroenteritis who was found to have severe HPVG on CT imaging.

## Case presentation

A 17-year-old female presented to the pediatric emergency department with a primary complaint of abdominal pain of six days duration. On days one to three of her illness she reported multiple episodes of nausea, non-bloody/non-bilious vomiting, non-bloody/non-melanotic diarrhea, and diffuse abdominal discomfort with distention. Over days three and four her nausea and abdominal pain marginally resolved but the diarrhea persisted. On days five to six, she noted resumption of nausea, worsening diarrhea, and severe worsening of diffuse abdominal pain. Her past medical history was non-contributory. She had no history of recent travel, recent illness, or change to her dietary habits.

Admission vital signs were heart rate 68/min, respiratory rate 17/min, blood pressure 100/53 mm Hg, and oxygen saturation of 99% on room air. Physical exam revealed a well-nourished patient in moderate discomfort with dry mucous membranes, abdominal distention, and tenderness in the left upper, left lower and right lower quadrants with point tenderness at McBurney’s point. There were no peritoneal signs or CVA tenderness. Neurologic exam was normal.

Laboratory investigation revealed a white blood cell (WBC) count of 8.2 k/uL [reference range (RR) 4.4- 10.5 k/uL] with otherwise unremarkable hemoglobin and hematocrit, basic metabolic panel, liver function panel, lipase, coagulation studies, and hepatitis panel. Her erythrocyte sedimentation rate (ESR) was within normal limits but her c-reactive protein (CRP) was mildly elevated to 6.8 mg/L [RR <5.1 mg/L]. Urinalysis was normal. Blood cultures were eventually negative.

**Figure 1:**
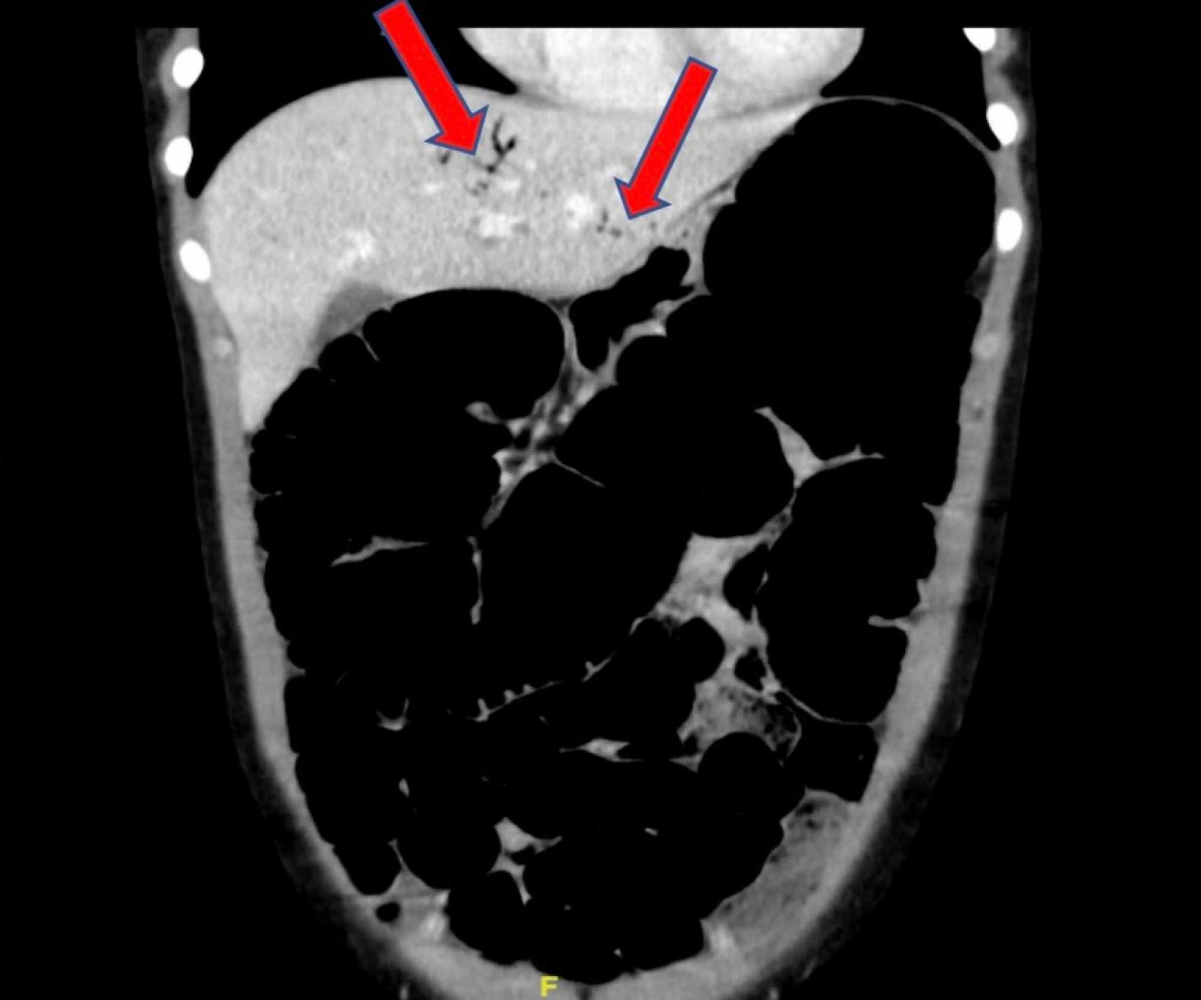
Coronal view CT scan of the abdomen showing marked hepatic venous portal gas

**Figure 2:**
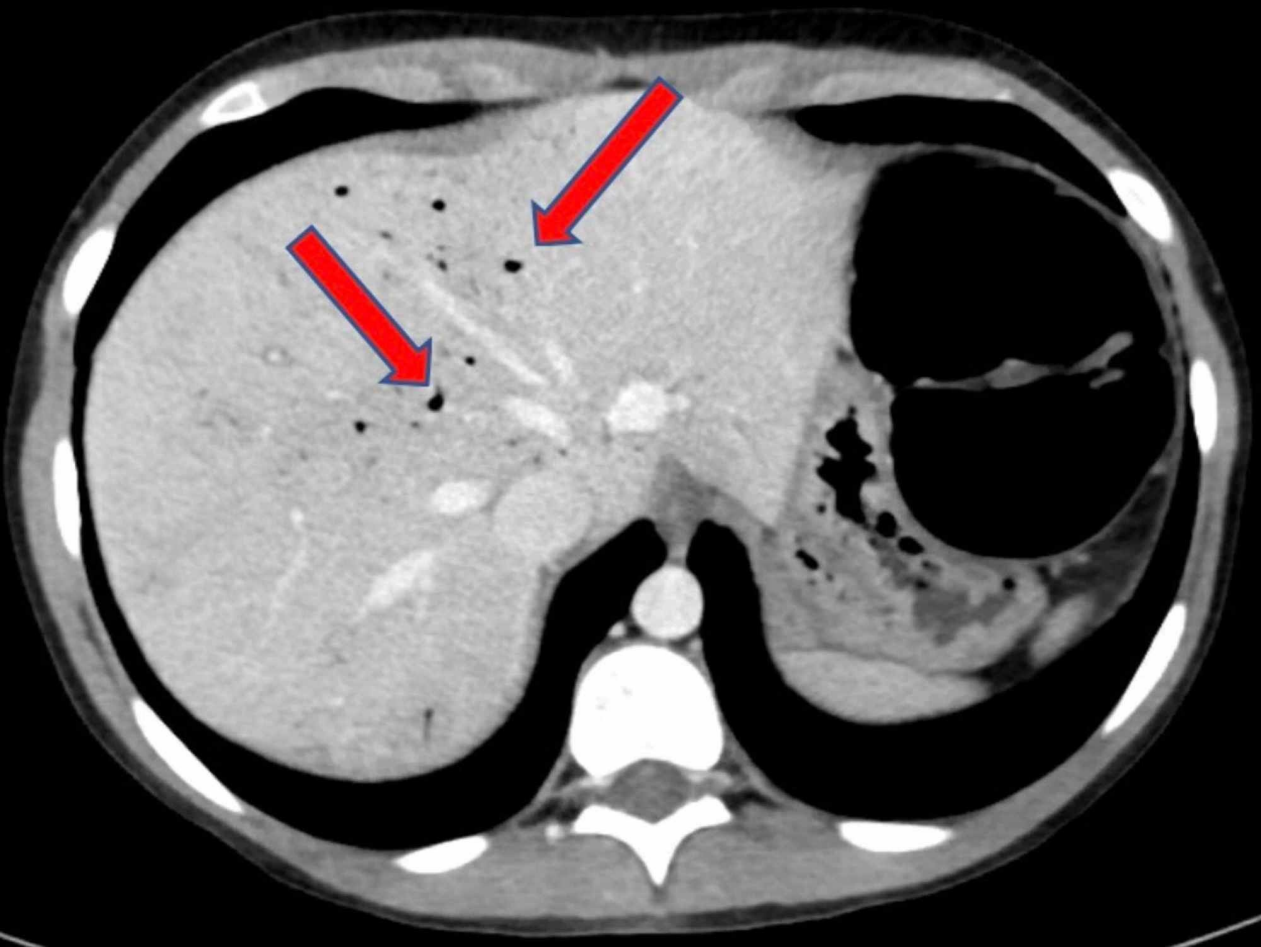
Transverse view CT scan of the abdomen showing marked hepatic venous portal gas

Given her age and vague localization of pain, an abdominal US was performed to investigate for appendicitis. Results were non-diagnostic. She subsequently received an abdominal CT scan which revealed dilation of the colon with fecalization of small bowel suggestive of low-grade obstruction/ileus. There was also associated significant portal venous gas consistent with severe bowel mucosal injury (Figures 1, 2). There was no discrete peritoneal fluid collection, focal bowel wall thickening, or intraperitoneal air.

The differential diagnosis of HPVG includes conditions listed in Table 1.

 

**Table 1:** Differential diagnosis of HPVG gastroenteritis References: [2,3,5-7]. HPVG = hepatic portal venous gas.

Differential diagnosis
Appendicitis
Pancreatitis
Viral gastroenteritis
Cholecystitis
Bowel necrosis
Bowel obstruction
Intra-abdominal abscess
Inflammatory bowel disease
Gastric ulcer
Intraperitoneal tumor

The patient received intravenous fluids, ceftriaxone and metronidazole, analgesics and was admitted to the ICU for serial abdominal exams. During the hospitalization she had voluminous diarrhea and passage of flatus. After 48 hours she was clinically improved and was discharged home to follow up with the gastroenterology service. Her stool culture was eventually positive for EPEC.

## Discussion

HPVG is characterized by branching radiolucencies within 2 cm of the liver capsule [7]. It is relatively uncommon in the pediatric population, but with the advent of more sensitive US and CT, detection frequency of incidental and clinically benign HPVG has increased [2,8]. Though the exact means by which gas enters the portal venous systems has not been elucidated, two physiologic mechanisms have been proposed: (1) gas-producing organisms entering/proliferating within the portal venous system; and (2) damage to the intestinal mucosa resulting in translocation of intestinal gas from the lumen of the bowel into the portal venous system [3]. Our case is the first reported association of bacterial EPEC gastroenteritis resulting in HPVG.

Regarding treatment, the radiographic identification of HPVG alone should not always prompt immediate surgical intervention. Rather, careful consideration of disease process and clinical presentation should help guide the decision to pursue clinical or surgical management [2]. Since invasive bacterial infection can be associated with this condition, empiric antibiotic therapy pending blood culture and improved clinical status are considerations. However, we found no standard recommendation for initiating empiric antibiotic therapy at the time of presentation to HPVG patients. Lastly, there is a paucity of literature commenting on the role of repeat imaging in cases of HPVG. It should certainly be a consideration based on the underlying etiology and degree of clinical compromise exhibited.

## Conclusions

Historically, HPVG has been associated with catastrophic clinical outcomes. With the advent of modern imaging modalities, there will likely continue to be an increase in the number of incidentally identified cases. Our case demonstrates that non-invasive bacterial enteritis should be considered a possible etiology for HPVG. Stool cultures and/or polymerase chain reaction (PCR) testing can be considered if the patient is having loose stool in the setting of HPVG to survey for invasive pathogens. Until more clear data is presented within the medical literature, patients with HPVG should continue to be admitted for monitoring to determine the scope and progression of disease.
